# Analysis of rare coding variants in 470,000 exome-sequenced subjects characterises contributions to risk of type 2 diabetes

**DOI:** 10.1371/journal.pone.0311827

**Published:** 2024-12-12

**Authors:** David Curtis

**Affiliations:** UCL, UCL Genetics Institute, London, United Kingdom; Odense University Hospital, DENMARK

## Abstract

**Aims:**

To follow up results from an earlier study using an extended sample of 470,000 exome-sequenced subjects to identify genes associated with type 2 diabetes (T2D) and to characterise the distribution of rare variants in these genes.

**Materials and methods:**

Exome sequence data for 470,000 UK Biobank participants was analysed using a combined phenotype for T2D obtained from diagnostic and prescription data. Gene-wise weighted burden analysis of rare coding variants in the new cohort of 270,000 samples was carried out for the 32 genes previously significant with uncorrected p < 0.001 along with 7 other genes previously implicated in T2D. Follow-up studies of *GCK*, *GIGYF1*, *HNF1A* and *HNF4A* used the full sample of 470,000 to investigate the effects of different categories of variant.

**Results:**

No novel genes were identified as exome wide significant. Rare loss of function (LOF) variants in *GCK* exerted a very large effect on T2D risk but more common (though still very rare) nonsynonymous variants classified as probably damaging by PolyPhen on average approximately doubled risk. Rare variants in the other three genes also had large effects on risk.

**Conclusions:**

In spite of the very large sample size, no novel genes are implicated. Coding variants with an identifiable effect are collectively too rare be generally useful for guiding treatment choices for most patients. The finding that some nonsynonymous variants in *GCK* affect T2D risk is novel but not unexpected and does not have obvious practical implications. This research has been conducted using the UK Biobank Resource.

## Introduction

A previous study carrying out gene-based weighed burden analysis of rare coding variants using 200,000 exome-sequenced UK Biobank participants identified three genes associated with type 2 diabetes (T2D) at exome-wide significance, *GCK*, *HNF4A* and *GIGYF1* [1]. While *GCK*, *HNF4A* were already well-recognised as causes of maturity onset diabetes of the young (MODY), the implication of *GIGYF1* was novel though was quickly confirmed in another study which had access to sequence data from 379,000 UK Biobank participants [2–4]. Although these three were the only genes which achieved exome-wide significance, a total of 32 genes were significant with an uncorrected p value < 0.001 whereas, given that there were 20,384 informative genes, only 20 would be expected by chance. Additionally, a number of these genes appeared to be of potential interest from a biological point of view. Of note, a number of other genes with well-established roles in T2D failed to produce strong evidence of association using the weighted burden analysis, consisting of *HNF1A*, *HNF1B*, *ABCC8*, *INSR*, *MC4R*, *SLC30A8* and *PAM*.

Subsequently, rare variant analyses using multiple different phenotypes were carried out in larger numbers of exome sequenced participants from the same UK Biobank cohort and some of the phenotypes studied included T2D and related conditions [5, 6].

Exome sequence data for a full set of 470,000 participants has now been made more widely available and the current study carried out weighted burden analysis in the new samples of the genes significant at p < 0.001 in the previous study, along with the other T2D implicated genes mentioned above. This study aimed to test for evidence of association and to compare results with those obtained from the multiple phenotype studies referred to above, as well as to characterise the effects of different categories of coding variant on risk in implicated genes. The purpose of this study was to use the 270,000 newly available exomes to test whether some of the genes which had produced results which were not significant after correction for multiple testing in the earlier study might yield evidence for association with the new sample. Additionally, having the larger sample of 470,000 would mean that it would be possible to more accurately model the effects on disease risk of different categories of variant in the associated genes.

## Materials and methods

The methods used were essentially the same as those described previously and are briefly repeated here for the reader’s convenience.

UK Biobank participants are volunteers intended to be broadly representative of the UK population and are not selected on the basis of having any health condition. UK Biobank had obtained ethics approval from the North West Multi-centre Research Ethics Committee which covers the UK (approval number: 11/NW/0382) and had obtained written informed consent from all participants. The UK Biobank approved an application for use of the data (ID 51119) and ethics approval for the analyses was obtained from the UCL Research Ethics Committee (11527/001). The data was accessed most recently on October 12 2023. There was no information which could be used to identify individual subjects. No subjects were minors. The UK Biobank Research Analysis Platform was used to access the Final Release Population level exome OQFE variants in PLINK format for 469,818 exomes which had been produced at the Regeneron Genetics Center using the protocols described here: https://dnanexus.gitbook.io/uk-biobank-rap/science-corner/whole-exome-sequencing-oqfe-protocol/protocol-for-processing-ukb-whole-exome-sequencing-data-sets [6]. All variants were then annotated using the standard software packages VEP, PolyPhen and SIFT [7–9]. To obtain population principal components reflecting ancestry, version 2.0 of *plink* (https://www.cog-genomics.org/plink/2.0/) was run with the options—*maf 0*.*1*—*pca 20 approx* [10, 11].

The T2D phenotype was defined in the same way as previously and was determined from three sources in the dataset: self-reported diabetes or type 2 diabetes (but not type 1 or gestational diabetes); reporting taking any of a list of named medications commonly used to treat T2D in the UK (https://www.diabetes.co.uk/Diabetes-drugs.html); having an ICD10 code for non-insulin-dependent diabetes mellitus in hospital records or as a cause of death [1]. Subjects in any of these categories were deemed to be cases while all other subjects were taken to be controls. In the primary analyses to implicate specific genes, attention was restricted to participants not included in the earlier study, consisting of 19,701 cases and 249,581 controls. For the subsequent analyses using the whole sample there were 33,629 cases and 436,136 controls.

The SCOREASSOC program was used to carry out a weighted burden analysis to test whether, in each gene, sequence variants which were rarer and/or predicted to have more severe functional effects occurred more commonly in cases than controls [12–14]. Attention was restricted to rare variants with minor allele frequency (MAF) < = 0.01 in cases or controls or both. As previously described, variants were weighted by overall MAF so that variants with MAF > = 0.01 were given a weight of 1 while very rare variants with MAF close to zero were given a weight of 10. Variants were also weighted according to their functional annotation using the GENEVARASSOC program, which was used to generate the input files for weighted burden analysis by SCOREASSOC. Variants predicted to cause complete loss of function (LOF) of the gene were assigned a weight of 100. Nonsynonymous variants were assigned a weight of 5 but if PolyPhen annotated them as possibly or probably damaging then 5 or 10 was added to this and if SIFT annotated them as deleterious then 20 was added. The full set of weights and categories is displayed in Table 1 of the previous study [1]. The weighting scheme had been devised to be broadly concordant with the observed effects of variants of different annotations and allele frequencies, as detailed in an earlier report [15]. As described previously, the weight due to MAF and the weight due to functional annotation were multiplied together to provide an overall weight for each variant. Variants were excluded if there were more than 10% of genotypes missing in the controls and cases or if the heterozygote count was smaller than both homozygote counts in controls and cases. If a subject was not genotyped for a variant then they were assigned the subject-wise average score for that variant. For each subject a gene-wise weighted burden score was derived as the sum of the variant-wise weights, each multiplied by the number of alleles of the variant which the given subject possessed.

**Table 1:** Results of gene-wise weighted burden analysis of rare variants in the original sample of 200,000 participants, the new sample of 270,000 and, for genes of interest, in the combined sample of 470,000.

Symbol	SLP in original sample	Name	SLP in new sample	SLP in combined sample
*GCK*	22.25	Glucokinase	12.09	32.11
*HNF4A*	6.82	Hepatocyte Nuclear Factor 4 Alpha	3.81	9.39
*GIGYF1*	6.22	GRB10 Interacting GYF Protein 1	2.42	7.58
*ZNF620*	3.78	Zinc Finger Protein 620	0.50	
*RAI2*	3.74	Retinoic Acid Induced 2	-0.03	
*TM4SF20*	3.65	Transmembrane 4 L Six Family Member 20	0.12	
*ALAD*	3.63	Aminolevulinate Dehydratase	1.25	
*PPARG*	3.45	Peroxisome Proliferator Activated Receptor Gamma	0.94	
*LOC105370752*	3.42	Uncharacterized LOC105370752	0.23	
*KLHL11*	3.35	Kelch Like Family Member 11	0.59	
*HMGXB4*	3.35	HMG-Box Containing 4	2.30	
*MIR6825*	3.31	MicroRNA 6825	0.00	
*TAZ*	3.30	Tafazzin	0.63	
*WDR33*	3.25	WD Repeat Domain 33	-0.23	
*HECTD1*	3.24	HECT Domain E3 Ubiquitin Protein Ligase 1	0.28	
*ZNF571-AS1*	3.23	ZNF571 Antisense RNA 1	-0.15	
*GYG1*	3.22	Glycogenin 1	-0.09	
*APTX*	3.20	Aprataxin	0.62	
*KCNK15*	3.19	Potassium Two Pore Domain Channel Subfamily K Member 15	-0.05	
*XPO1*	3.19	Exportin 1	1.24	
*PKD1*	3.10	Polycystin 1, Transient Receptor Potential Channel Interacting	0.11	
*ZNF763*	-3.01	Zinc Finger Protein 763	1.24	
*COA5*	-3.05	Cytochrome C Oxidase Assembly Factor 5	-0.11	
*GHRL*	-3.15	Ghrelin And Obestatin Prepropeptide	0.22	
*DEUP1*	-3.20	Deuterosome Assembly Protein 1	0.39	
*C7orf50*	-3.22	Chromosome 7 Open Reading Frame 50	-0.54	
*MFSD12*	-3.34	Major Facilitator Superfamily Domain Containing 12	-0.75	
*C19orf73*	-3.34	Chromosome 19 Open Reading Frame 73	-0.01	
*ATXN1L*	-3.35	Ataxin 1 Like	-1.08	
*EML4*	-3.58	EMAP Like 4	0.21	
*DLEC1*	-3.72	DLEC1 Cilia And Flagella Associated Protein	-0.16	
*RPS5*	-3.76	Ribosomal Protein S5	0.27	
*HNF1A*	1.66	HNF1 Homeobox A	7.17	7.98
*HNF1B*	-0.29	HNF1 Homeobox B	-0.21	
*ABCC8*	1.94	ATP Binding Cassette Subfamily C Member 8	2.44	
*INSR*	-0.25	Insulin Receptor	0.25	
*MC4R*	1.41	Melanocortin 4 Receptor	1.37	
*SLC30A8*	-2.64	Solute Carrier Family 30 Member 8	-1.16	
*PAM*	0.19	Peptidylglycine Alpha-amidating Monooxygenase	1.30	

Analyses were restricted to the 32 genes significant at p < 0.001 in the previous study along with the other 7 listed above as being previously implicated in T2D. For each gene, logistic regression analysis was carried out with T2D as the dependent variable including the first 20 population principal components and sex as covariates and a likelihood ratio test was performed comparing the likelihoods of the models with and without the gene-wise burden score. This is a test for association between the gene-wise burden score and caseness and the statistical significance was summarised as a signed log p value (SLP), which is the log base 10 of the p value given a positive sign if the score is higher in cases and negative if it is higher in controls. Since only 39 genes were analysed and each gene was subjected to a single test, in total only 39 tests were performed in the new samples. This means that after a Bonferroni correction for multiple testing a gene could be declared statistically significant if it achieved an SLP with absolute value greater than -log10(0.05/39) = 2.89 using the new samples.

Follow-up analyses were performed on all genes individually achieving this significance level and also *GIGYF1*, because this gene had reached conventional levels of exome-wide statistical significance in the earlier study of this dataset. For this subset of genes the weighted burden analysis described above was repeated using the whole sample of 33,629 cases and 436,136 controls. Additionally, for each subject a count was obtained of the number of variants they carried falling into particular broad annotation categories, such as LOF, protein altering, etc. The full list of these categories is shown in S1 Table. These counts were entered into a multiple logistic regression analysis with T2D as the dependent variable and again including sex and 20 principal components as covariates in order to elucidate the contribution of different types of variant to the overall evidence for association. The odds ratios (ORs) associated with each category were estimated along with their standard errors and the Wald statistic was used to obtain a p value. This p value was converted to an SLP, again with the sign being positive if the OR was greater than 1, indicating that variants in that category tended to increase risk.

Data manipulation and statistical analyses were performed using GENEVARASSOC, SCOREASSOC and R [13, 14, 16].

## Results

Table 1 shows the results of the primary analysis, presenting the SLPs obtained in the previous study along with those obtained in the new sample. Of the genes showing evidence for association in the previous study, only *GCK* (SLP = 12.09) and *HNF4A* (SLP = 3.81) are formally significant after correction for multiple testing, while *GIGYF1* yields SLP = 2.42 and none of the other genes previously with p < 0.001 shows evidence for association in the new sample. Of the 7 genes implicated in T2D in earlier studies, only *HNF1A* (SLP = 7.17) is formally statistically significant.

The four genes named above were carried forward for secondary analyses. The original study considered 20,384 genes, meaning that for a gene-wise result to be considered exome-wide significant the magnitude of the SLP obtained should exceed -log10(0.05/20384) = 5.61. For the four genes carried forward, the results of weighted burden analysis in the entire sample of 33,629 cases and 436,136 controls are also shown in Table 1 and it can be seen that all four of these genes produce results which would be regarded as exome-wide significant in the full sample.

In order to gain insights into the effects of different categories of variant within these four genes of interest, counts for variants of each category in each subject were entered into multiple logistic regression analysis along with sex and 20 principal components as covariates. These results are shown in Table 2 and are summarised briefly as follows.

**Table 2:** Results from logistic regression analysis including principal components and sex as covariates showing the contribution different categories of variant within each gene make to risk of hyperlipidaemia. Odds ratios for each category are estimated and the strength of evidence for an effect is expressed as the SLP.

**2A:** Results for *GCK*							
**Variant category**	**Number of separate variants**	**Total count in controls**	**Mean count in controls**	**Total count in cases**	**Mean count in cases**	**OR (95% confidence interval)**	**SLP**
Intronic, etc	722	22545	0.051692	2454	0.072973	1.03 (0.98–1.08)	0.61
5 prime UTR	84	2185	0.005011	311	0.009251	1.05 (0.92–1.19)	0.32
Synonymous	149	10442	0.023942	795	0.023640	0.93 (0.86–1.00)	-1.32
Splice region	55	4240	0.009722	322	0.009575	1.05 (0.93–1.17)	0.35
3 prime UTR	41	3647	0.008363	245	0.007286	0.91 (0.80–1.04)	-0.81
Protein altering	218	1725	0.003955	233	0.006929	1.01 (0.84–1.23)	0.05
Indel, etc	3	9	0.000021	2	0.000059	2.89 (0.60–13.93)	0.74
LOF	25	15	0.000034	28	0.000833	24.41 (12.61–47.25)	21.35
SIFT deleterious	116	627	0.001438	98	0.002914	1.31 (0.90–1.91)	0.81
PolyPhen possibly damaging	36	206	0.000472	25	0.000743	1.23 (0.73–2.06)	0.37
PolyPhen probably damaging	84	304	0.000697	69	0.002052	2.45 (1.62–3.70)	4.84
**2B:** Results for *GIGFY1*							
**Variant category**	**Number of separate variants**	**Total count in controls**	**Mean count in controls**	**Total count in cases**	**Mean count in cases**	**OR (95% confidence interval)**	**SLP**
Intronic, etc	1419	54973	0.126046	4845	0.144073	1.01 (0.98–1.04)	0.36
5 prime UTR	41	421	0.000965	47	0.001399	0.9 (0.66–1.23)	-0.31
Synonymous	451	11251	0.025797	982	0.029203	0.97 (0.91–1.04)	-0.42
Splice region	148	8098	0.018568	825	0.024533	1.01 (0.94–1.08)	0.11
3 prime UTR	63	4903	0.011242	356	0.010578	0.88 (0.78–0.98)	-1.76
Protein altering	843	21609	0.049546	2084	0.061975	1 (0.94–1.07)	0.04
Indel, etc	47	1186	0.002720	91	0.002706	1.01 (0.82–1.26)	0.05
LOF	83	135	0.000310	39	0.001160	3.44 (2.38–4.96)	10.78
SIFT deleterious	443	3164	0.007255	285	0.008475	1.01 (0.88–1.17)	0.06
PolyPhen possibly damaging	162	5444	0.012482	395	0.011746	1.00 (0.88–1.13)	-0.01
PolyPhen probably damaging	244	2922	0.006700	283	0.008415	1.21 (1.04–1.41)	1.97
**2C:** Results for *HNF1A*							
**Variant category**	**Number of separate variants**	**Total count in controls**	**Mean count in controls**	**Total count in cases**	**Mean count in cases**	**OR (95% confidence interval)**	**SLP**
Intronic, etc	654	22154	0.050795	2087	0.062058	0.98 (0.93–1.03)	-0.47
5 prime UTR	69	992	0.002274	136	0.004055	1.30 (1.08–1.56)	2.31
Synonymous	223	3606	0.008268	369	0.010973	1.03 (0.92–1.15)	0.22
Splice region	48	1304	0.002990	198	0.005888	1.09 (0.93–1.28)	0.56
3 prime UTR	48	775	0.001777	92	0.002736	1.06 (0.85–1.34)	0.23
Protein altering	401	6623	0.015186	675	0.020072	1.09 (0.98–1.22)	0.94
Indel, etc	6	19	0.000044	4	0.000119	3.12 (1.03–9.44)	1.11
LOF	18	71	0.000163	24	0.000714	4.88 (3.01–7.88)	10.36
SIFT deleterious	228	3004	0.006888	286	0.008505	0.95 (0.78–1.16)	-0.21
PolyPhen possibly damaging	96	1034	0.002371	98	0.002914	0.96 (0.75–1.23)	-0.12
PolyPhen probably damaging	126	1028	0.002357	114	0.003390	1.33 (1.02–1.72)	1.52
**2D:** Results for *HNF4A*							
**Variant category**	**Number of separate variants**	**Total count in controls**	**Mean count in controls**	**Total count in cases**	**Mean count in cases**	**OR (95% confidence interval)**	**SLP**
Intronic, etc	935	27523	0.063108	2774	0.082483	1 (0.96–1.04)	0.01
5 prime UTR	62	795	0.001823	57	0.001682	0.81 (0.61–1.07)	-0.87
Synonymous	192	11920	0.027332	1039	0.030897	1.02 (0.96–1.09)	0.34
Splice region	51	2656	0.006090	319	0.009488	1.05 (0.93–1.20)	0.38
3 prime UTR	54	1089	0.002497	119	0.003541	0.9 (0.73–1.09)	-0.57
Protein altering	301	3225	0.007395	399	0.011865	1.08 (0.93–1.25)	0.49
Indel, etc	2	8	0.000018	5	0.000149	9.76 (3.09–30.86)	2.83
LOF	9	9	0.000021	1	0.000030	1.78 (0.27–11.70)	0.28
SIFT deleterious	126	1400	0.003210	173	0.005144	1.34 (0.96–1.86)	1.09
PolyPhen possibly damaging	40	738	0.001692	68	0.002022	0.88 (0.59–1.29)	-0.3
PolyPhen probably damaging	59	326	0.000747	69	0.002052	1.87 (1.26–2.78)	2.79

Table 2A shows that LOF variants in *GCK* exert a substantial risk of T2D, with OR over 20, but that nonsynonymous variants classified as probably damaging by PolyPhen also increase risk, with OR estimated as 2.45. Variants in this latter category are observed 363 times in the sample of 470,000 participants, so occur in less than 1 in 1,000 people, whereas the LOF variants are rarer still, being seen only 43 times.

Table 2B shows that LOF variants in *GIGYF1* are slightly commoner than in *GCK*, being seen 174 times, although they remain extremely rare. They have a more moderate effect on risk, with OR estimated as only 3.44. No other categories of variant have a clear effect on risk, though it is possible that variants classified has probably damaging by PolyPhen (SLP = 1.97) have a small effect (OR = 1.21).

Table 2C shows that LOF variants in *HNF1A* increase risk with OR = 4.88, but there may also be a modest effect of 5-prime UTR variants (SLP = 2.31, OR = 1.30) and/or variants classified as probably damaging by PolyPhen (SLP = 1.52, OR = 1.33).

Table 2D shows that LOF variants in HNF4A are extremely rare and do not have a detectable effect. Rather, the signal implicating this gene seems to come from variants classified as probably damaging by PolyPhen (SLP = 2.79, OR = 1.87) and two indel variants. These two variants consisted of 20:44428418GCCAACACAATGC>G (rs1349603952), observed in 4 controls and 2 cases, and 20:44424132A>AGCT (rs776489992), observed in 4 controls and 3 cases. Malacards lists 4 entries for rs776489992, with phenotypes MODY, MODY Type 1, T2D and Fanconi Renotubular Syndrome 4 with MODY (https://www.malacards.org/search/results?query=rs776489992). However there are no previous reports for rs1349603952.

## Discussion

These analyses provide very strong support for *GCK* as a risk gene for T2D while three other previously identified genes also achieve conventional levels of significance: *GIGYF1*, *HNF1A* and *HNF4A*. However, no novel genes are implicated. As mentioned previously, this dataset has been used for analyses of multiple phenotypes including some relating to T2D. which we can refer to as the Regeneron and AstraZeneca studies (Backman et al., 2021; Wang et al., 2021). The Regeneron study carried out a variety of single variant and gene-wise burden tests on 3,994 health-related traits to produce a total of about 2.3 billion tests, yielding a critical p value of 2.18e-11 (corresponding to SLP = 10.66), and reported 8,865 significant associations which are presented in their Supplementary Data 2 (Backman et al., 2021). 64 associations were reported between *GCK* and diabetes or related phenotypes, with the most significant being with glycated haemoglobin HbA1c at p = 4.98e-22, equivalent to SLP = 21.30, whereas in the current study *GCK* yields SLP = 32.11. *GIGYF1* was associated with T2D at SLP = 12.34 and *HNF1A* with T2D at SLP = 12.58. However no association with a diabetes-related phenotype was reported for *HNF4A*, although it was associated with levels of sex hormone binding globulin (SHBG) at SLP = 41.85. For the AstraZeneca study, all gene-wise and variant-wise associations with 17,361 binary and 1,419 quantitative phenotypes are reported on the AstraZeneca PheWAS Portal at *https*:*//azphewas*.*com* (Wang et al., 2021). This was accessed to find the most significant p value for any analysis of each of these genes with the phenotype "Union#E11#Type 2 diabetes mellitus" and Table 3 shows the results obtained compared with those for the current study. It can be seen that the current study again produces stronger evidence for association with *GCK*, with SLP = 32.11 versus SLP = 23.10 for the AstraZeneca study, whereas for the other three genes the strength of evidence for association is fairly similar between the two studies.

**Table 3:** Comparison of results from current study to those reported for the AstraZeneca study. The results for the AstraZeneca study are displayed as the equivalent SLP for the most significant result reported for that gene with the phenotype "Union#E11#Type 2 diabetes mellitus".

Gene	SLP for combined sample in current study	SLP for AstraZeneca study
*GCK*	32.11	23.10
*GIGYF1*	7.58	8.77
*HNF1A*	7.98	6.85
*HNF4A*	9.39	9.37

The fact that current study finds stronger evidence for association of *GCK* relative to the other analyses may reflect the fact that, for this gene, the pattern of effects due to different variant types does resemble the model which is assumed for the weighted burden analysis, with strong effects due to LOF variants and more moderate effects due to some nonsynonymous variants. However for the other three genes this pattern is not seen and hence for them the weighted burden analysis does not have advantages over more conventional variant pooling analyses. In a subsequent study which used a wide variety of different methods to predict the effects of nonsynonymous variants, it was observed that other predictors would produce stronger evidence for effects of nonsynonymous variants in these genes [17]. However using a variety of different predictors would require correction for multiple testing and so was not thought appropriate for the current study, which aimed simply to obtain evidence for assocation at the level of the gene.

It is of interest to note that the evidence in favour of the association with T2D risk is considerably higher for *GCK* than for the other genes, and likewise the effect size of implicated variants is larger. It is tempting to speculate that this relates to the molecular mechanisms underlying the observed association. The product of *GCK*, glucokinase, is a low-affinity hexose kinase which acts as the rate limiting enzyme for glycolysis in pancreatic islet cells, as well as in some hepatocytes and neurons, meaning that it can be used by these cells as an indicator of blood glucose levels [18, 19]. Thus, impaired functioning of glucokinase is expected to lead to reduced sensitivity to higher glucose levels and hence inadequate glycaemic control. By contrast, *GIGYF1*, *HNF1A* and *HNF4A* are involved with lower level cellular processes which have a less immediate impact in terms of producing diabetes as a phenotype. The product of *GIGFY1* binds to Grb10, a protein which regulates the response to insulin-like growth factor receptor signalling and it is associated with a number of different phenotypes in addition to T2D, including lipid-related phenotypes, education score, cognitive function and cystatin C levels [6, 20–22]. The products of *HNF1A* and *HNF4A* are transcription factors affecting the expression of large numbers of other genes and influencing development of the liver and pancreas [23]. Biallelic variants in *HNF1A* can cause hepatocellular adenomas, while variants in *HNF4A* can cause Fanconi renotubular syndrome and are associated with SHBG levels [6, 24, 25]. The fact that *GCK* has such a direct effect on contributing to the control of glucose levels may explain in part why LOF variants in it have a larger effect on the T2D phenotype than for other genes.

The emphasis of the current study is to detect and characterise association at the level of the gene and of categories of variant within the gene, even though many of the variants concerned are too rare to be tested individually. However it is recognised that within an associated category there will be some variants having an effect on risk and others which do not. When the same variant is observed in multiple individuals then it would be possible to attempt to model the individual effect of such a variant in terms of its estimated odds ratio or penetrance, as has been carried out using variants designated as pathogenic in these genes in a subsample of the UK Biobank dataset [26]. The availability of the AstraZeneca PheWAS Portal at *https*:*//azphewas*.*com* means that for any such variant one and any studied phenotype one can obtain the variant counts in controls and cases in order to estimate the odds ratio and/or penetrance.

It could be argued that the work presented here highlights some of the limitations as well as strengths of analysing rare coding variants identified in exome-sequencing studies of large population cohorts. Because of the high prevalence of T2D, many thousands of cases are available for study but, as the results show, only a small fraction of these cases carry a variant in a category which can be identified as impacting risk. A number of genes which had previously been implicated in targeted studies do not in the current study yield evidence at conventional levels of statistical significance after correction for multiple testing. Although T2D has a high prevalence, many other clinically important phenotypes have a substantial genetic contribution to risk but with a lower prevalence and there would be insufficient case numbers present in an unselected cohort for similar approaches to be likely to yield any convincing novel rare variant associations. In order to identify genes involved in such conditions it would be necessary to carry out studies involving specifically recruited cases, perhaps also focusing on those from densely affected families where large-effect variants may be active. To support such initiatives, it would be helpful to strengthen methods to incorporate existing samples as controls rather than requiring that a matching set of controls be recruited and sequenced for each new set of cases. Using existing samples as controls has been helpful in other sequencing studies but requires careful alignment of methodologies to minimise artefacts [27].

If adequately sized samples are used, exome sequencing studies can identify genes in which damaging variants have large effects on risk of particular phenotypes. The main value of such studies is to implicate specific genes, and hence their protein products, as impacting the phenotype. This may ultimately lead to a better understanding of the molecular pathways involved in pathogenesis. However, because for non-Mendelian diseases identifiable variants are only seen in a very small proportion of cases, typically fewer than 1%, such approaches seem unlikely to be helpful to guide individual treatment interventions in most situations. The vast majority of patients would not carry a variant which could be clearly identified as causal, and even if such a variant were encountered this might not automatically have clear implications for treatment choices. Taking the results obtained from the current study as an example, fewer than 1% of cases have a variant in one of the four identified genes which would be classified as having a pathogenic effect. A recent review provides an account of the variations in clinical course and responses to treatment in individuals carrying pathogenic variants in *GCK*, *HNF1A* or *HNF4A* and hence identifying these cases might provide some therapeutic benefit [28]. However for most patients with T2D, genetic screening would not be expected to produce actionable results.

Exome-sequencing studies to date, including the current one, now fairly consistently show that the category of variant having the highest identifiable impact on phenotype consists of those variants which are predicted to cause loss of function of the gene, or haploinsufficiency. This is not to say that individual variants in other categories might not have larger effects, and of course the literature is replete with examples of these. However, in a situation where individual variants are extremely rare, as is expected for those with large effect, it becomes necessary to pool variants together in some form of burden analysis and currently available methods for prediction of impact of non-LOF variants on the function of the gene and/or protein product are not able to reliably discriminate those which are pathogenic from those without major effect. If for a given gene-phenotype pair we can discover that that LOF variants have a particular effect on increasing or decreasing risk then this may provide an important endpoint in terms of improved insight into the molecular pathways involved in pathogenesis. For example, this might be sufficient to flag up the protein as a possible drug target. However it is possible to argue that additional useful information could be gained from more intensive investigations to elucidate the effects of other types of variant. For example, if one can find that non-synonymous variants affecting particular protein domains tend to show evidence of association this might yield a more sophisticated understanding of disease mechanisms which again might potentially be exploited therapeutically.

The present study confirms the role of four previously implicated genes in risk of T2D. It also demonstrates that nonsynonymous variants in *GCK* which PolyPhen annotates as probably damaging on average approximately double risk of T2D, although as these variants are still very rare this finding may not have much in the way of practical applications. The results show the distributions of different categories of variant in these genes in the general population. Overall, the study provides some insights into what can be achieved from the analysis of exome sequence data and into some limitations of such approaches.

## Supporting information

S1 Table The table shows the broad categories used for variant category specific analyses along with the annotations produced by VEP which were grouped into each category. (DOCX)
