# Supplementary material for: Analysis of rare coding variants in 470,000 exome-sequenced subjects characterises contributions to risk of type 2 diabetes
Source: PLoS One. 2024 Dec 12;19(12):e0311827. doi: 10.1371/journal.pone.0311827 (PMC11637267; doi:10.1371/journal.pone.0311827)
Supplement: S1 Table — (DOCX) [file pone.0311827.s001.docx]

**Analysis of rare coding variants in 470,000 exome-sequenced subjects characterises their contributions to risk of type 2 diabetes**

David Curtis

**Supplementary Table 1**

The table shows the broad categories used for variant category specific analyses along with the annotations produced by VEP which were grouped into each category.

| Category | VEP annotation |
| --- | --- |
| Intronic etc. | feature_truncation, regulatory_region_variant, feature_elongation, regulatory_region_amplification, regulatory_region_ablation, TF_binding_site_variant, TFBS_amplification, TFBS_ablation, downstream_gene_variant, upstream_gene_variant, non_coding_transcript_variant, NMD_transcript_variant, intron_variant, non_coding_transcript_exon_variant |
| Five prime UTR | 5_prime_UTR_variant |
| Synonymous | synonymous_variant |
| Splice region | splice_region_variant |
| Three prime UTR | 3_prime_UTR_variant |
| Protein altering | protein_altering_variant, missense_variant |
| Indel etc. | inframe_deletion, inframe_insertion, transcript_amplification |
| LOF | frameshift_variant, stop_gained , transcript_ablation, splice_donor_variant, splice_acceptor_variant |
| SIFT deleterious | deleterious |
| PolyPhen possibly damaging | possibly_damaging |
| PolyPhen probably damaging | probably_damaging |
